# Health-related quality of life in polycystic ovary syndrome patients: A systematic review 

**Published:** 2015-08

**Authors:** Seyed Abdolvahab Taghavi, Fatemeh Bazarganipour, Ali Montazeri, Anoshirvan Kazemnejad, Reza Chaman, Ahmad Khosravi

**Affiliations:** 1 *Mother and Child Welfare Research Center, Hormozgan University of Medical Sciences, Bandar Abbas, Iran.*; 2 *Mental Health Research Group, Health Metrics Research Center, Iranian Institute for Health Sciences Research, ACECR, Tehran, Iran.*; 3 *Department of Biostatistics, Tarbiat Modares University, Tehran, Iran.*; 4 *Department of Social Medicine, School of Medicine, Yasuj University of Medical Sciences, Yasuj, Iran. *; 5 *Center for Health Related Social and Behavioral Sciences Research, Shahroud University of Medical Sciences, Shahroud, Iran.*

**Keywords:** *Polycystic ovary syndrome*, *Quality of life*, *Questionnaire*, *Systematic review*

## Abstract

**Background:**

Increasing attention to the concept of polycystic ovary syndrome (PCOS) health-related quality of life has led to the development of tool that aims to measure this concept.

**Objective:**

The purpose of this study was to conduct a systematic review of psychometric properties of the PCOS health-related quality of life questionnaire.

**Materials and Methods:**

A search of database (Pubmed, PsychInfo, CINAHL, CENTRAL, Scopus and SID) from January1998 to December 2013 yielded 6152 references of which 27 papers remained after review of the titles and abstracts. The reviewers used structural tools to analyze the articles, critically appraise papers, and extract the data. Finally, eight papers met the full inclusion criteria.

**Results:**

Studies suggested that the PCOS health-related quality of life questionnaire (PCOSQ)/or its modified version (MPCOSQ) have partial known groups validity. The convergent/divergent validity of the questionnaire also was found to be relatively acceptable. The PCOSQ/MPCOSQ reached acceptable benchmarks for its reliability coefficients. Regarding structural validity, some studies suggested that the PCOSQ/MPCOSQ have an extra dimension (related to menstruation) in addition to its existing dimensions for original or modified versions.

**Conclusion:**

The PCOSQ/MPCOSQ showed acceptable content and construct validity, reliability and internal consistency. However, some other properties, particularly those related to factor and longitudinal validity, absolute error of measurement, minimal clinically important difference and responsiveness still need to be evaluated.

## Introduction

Polycystic ovary syndrome (PCOS) is the most common endocrine disorder in women of reproductive age. It is estimated that 5 to 10% of women suffer from the disease (1). The issue of the quality of life (QOL) of patients with PCOS is very often overlooked in the clinical practice (2). One reason for such limited focus on QOL in patients with PCOS might be the fact that while generic questionnaires exist to measure health-related quality of life (HRQOL), they do not have the ability to capture information on all of the important areas of well-being and performance of women with PCOS. For example, infertility and hirsutism can place a considerable strain on the emotional well-being and personal relationships of women with PCOS. It seems that the PCOS health-related quality of life questionnaire (PCOSQ) is the only specific instrument exists to measure QOL in this population (3).

PCOSQ is among well-developed disease specific tools that was developed by Cronin *et al. *(1998) (2). Cronin *et al*. used semi-structured interviews, a health-practitioner survey and conducted a literature review to identify 182 items potentially relevant to women with PCOS. One hundred patients with PCOS reviewed 182 items, decided what items cause problems and rated the importance of the items. The final PCOSQ includes 26 items that takes 10-15 minutes to fill out. A factor analysis guided the categorization of the most important items into five areas or domains including concerns about hirsutism, emotion, weight, infertility, and menstruation. The PCOSQ reliability is good (3-5), but its validity showed controversial results due to the absence of measuring acne. Thus, the PCOSQ was modified (MPCOSQ) by Barnard *et al.* (2007) and four questions were added to the PCOSQ in order to evaluate issues associated to acne (6).

It is only by synthesizing information from several studies that we can understand how a measurement tool performs across different contexts and applications. The synthesis of the collection of data is a mechanism to provide more stable estimates of measurement errors and benchmarks for change/outcomes. In fact, two systematic reviews have been conducted where in psychometric properties are described, but a rating list with explicit criteria for psychometric quality is lacking (7-8). None previous reviews have included a key element of systematic review and critical appraisal of the quality of individual studies. This may reflect inherent difficulties in performing the critical appraisal due to lack of instrumentation. A systematic review can overcome these problems. In a systematic review all available evidence is reviewed in a systematic, transparent and reproducible manner. This ensures that almost all relevant literature will be located and it enables the reader to gain insight into the methods used.

In the current review, we used a scale and interpretation guide for this purpose (9-10). Psychometric studies are important to establish measurement properties like the relative difficulty of items, reliability, validity, and responsiveness. In addition to psychometric properties, clinicians are concerned with issues on feasibility, floor/ceiling effects, availability of different language/cultural adaptations, and administration burden for themselves and their patients. When therapists try to integrate the PCOSQ/MPCOSQ into their clinical practice, they are concerned with the psychometric issues but also need information on their usefulness. The purpose of this study was to conduct a systematic review that would summarize the quality and content of current research regarding the psychometric properties of the PCOSQ/MPCOSQ.

## Materials and methods

**Search strategy**

We conducted a systematic review on published literature using Pubmed, PsychInfo, Cochrane Central Register of Controlled Trials (CENTRAL(, CINAHL, and Scopus, included papers written from January 1998 to December 2013. 

A combination of the following keywords were used to search all databases for eligible studies: “Quality of life, Health-related quality of life, Satisfaction, Health status, Questionnaire, Health status measurement, Quality of life questionnaire, Psychometric, Psychometrics, Reliability, validity, Validation” and “Polycystic ovary syndrome, Polycystic ovaries, PCOS, Polycystic ovarian syndrome”.

**Exclusion and inclusion criteria**

An article was accepted if it met the following inclusion criteria: reported on at least one psychometric property of the PCOSQ/MPCOSQ in patients with PCOS and was written in English.

**Quality assessment**

Following this, two reviewers independently evaluated an assigned subset of articles using previously developed data extraction forms and quality appraisal tools that were developed by MacDermid (9-10). 

After the independent evaluation, the reviewers met to discuss the articles. Each specific item on the quality appraisal tool was openly discussed to reach consensus. 

This process identified whether disagreements were related to facts or adherence to the defined standards. When no consensus was achieved, reviewers considered the default option to be lower than the scores. 

Inter-rater reliability on quality ratings was calculated based on pre-consensus scores of reviewers; the overall estimated kappa was 0.82, with agreement on individual items varying from 0.43 to 1.00.

Each paper’s score was converted into a percentage because one item was based on follow-up and some psychometric studies were cross-sectional, leaving unequal denominators for different studies. 

We ranked ordered studies on quality and considered this ranking when making conclusions and recommendations, although there was no formal mechanism to weight conclusions, based on the quality of the associated source document. Investigational review board approval was not required for this systematic review.

**Statistical analysis**

The psychometric properties of outcome measurement should be assessed by their face and content validity, construct validity, reliability, responsiveness, interpretability, and acceptability and responder burden (11). A full explanation of these concepts is beyond the scope of this paper and the reader is referred to Fitzpatrick *et al.* (11) or Norman and Streiner (12), however a brief definition of these psychometric criteria in the context of patient-based questionnaires is given in Appendix 1.

## Results

**Figure 1:**
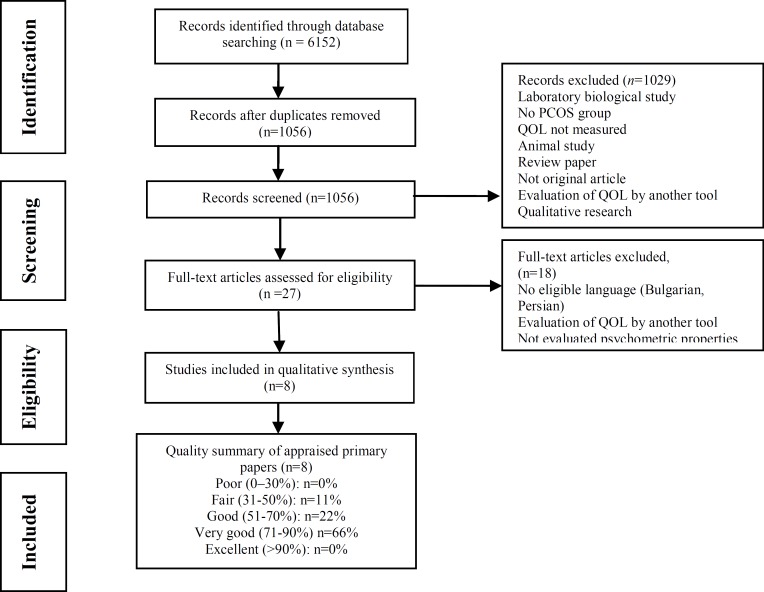
Flow diagram of study selection

In total, 8 studies were included in this systematic review. Figure 1 shows the flowchart of article selection according to the Preferred Reporting Items for Systematic Reviews and Meta-Analyses (PRISMA) statement (13).

**Quality assessment**

Quality of the individual studies was variable, ranging from 55% to 77%, with 55% of papers reaching or exceeding a score of 75% on the quality rating (Table1). The most common flaws observed in the psychometric articles were (1) not reporting specific scope of measurement, (2) inadequate sample size calculations/justification, and (3) absence of error estimates such as confidence intervals or standard error of measurement (SEM). A descriptive synthesis of the findings for psychometric properties across all identified studies is summarized in table 2. 

Due to the heterogeneity of study populations and properties evaluated, no meta-analyses were performed. Most studies addressed a spectrum of psychometric properties, but few were comprehensive.

The type of data collected during PCOSQ/MPCOSQ validation studies was typically comprised of less clinically useful data, like correlations indicating construct and convergent validity, versus more useful information, like known group differences that could be used as comparative data for clinical comparisons. 

Similarly, group reliability, such as intra-class correlation coefficients (ICCs), was reported more frequently rather than being more useful as indicators of absolute measurement error (like SEMs, mean retest differences, or minimal detectable change (MDC)).

**Psychometric properties of the PCOSQ/ MPCOSQ**

**1. Usefulness / Practicality (readability/ language and cultural applicability/administration burden)**

A number of papers have addressed issues around readability, usually in the context of language and cultural translations. Bazarganipour *et al.* (14) reported that the item of MPCOSQ "felt unsexy because of being overweight" had different meaning in Iranian culture. For that reason, it was changed to "felt not having sexy attractiveness because of being overweight" to adapt to the Iranian culture and make the questionnaire more understandable for this population. Moreover, they mentioned that almost all patients indicated that the questionnaire was easy to read and understand. Minor wording changes were done according to patient’s suggestions to improve clarity but not mentioned which item changes. Jedel *et al.* (15) ensured good readability of the Swedish version of the PCOSQ through a series of translating and back translating process. In the English version, 25% of patients mentioned that questions relating to their symptom of acne were missing from the PCOSQ and were worried by the question that asked about cancer. Seventeen percent of patients felt the questionnaire did not address their feelings of frustration about the lack of available information on PCOS (4). To our knowledge, there was no report related to specifically address or state how they measured the time taken to complete the PCOSQ/MPCOSQ. Although, Bazarganipour *et al*. (14) reported that the questionnaire completed in the waiting room and thus it seems did not add any additional time to the patient’s visit.

**2. Reliability**

**a. Internal consistency**

For all five subscales of the PCOSQ, internal consistency was acceptable (α ≥ 0.70) in three articles (4-5, 16), but two of the subscales did not demonstrate internal consistency in one study. Guyatt *et al.* (3) found Cronbach’s alpha levels for the menstruation subscale to be lower than acceptable level (α =0.62 at baseline and 0.54 at a 44-week follow up). McCook *et al.* (5) reported that the alpha levels of the emotion and menstruation subscales improved considerably when one item (worry about late period) was moved from the emotion to the menstruation subscale. For all six subscales of the MPCOSQ, internal consistency was acceptable (α ≥ 0.70) in all articles (6, 14). Moreover, Bazarganipour et al. (14) reported similar finding to McCook et al. (5) study. They mentioned that the alpha levels of the emotion and menstruation subscales improved considerably when one item (worry about late period) was moved from the emotion to the menstruation subscale.

**b. Test-retest reliability**

Jones *et al*. (4) found the PCOSQ to have acceptable test–retest reliability when re-administering the questionnaire after a 3-6 day interval. Correlation coefficients ranged from 0.89 to 0.95 for all subscales and these were found to be statistically significant (P<0.001 for all domains). Jedel *et al.* (15) found the PCOSQ to have acceptable test–retest reliability when re-administering the questionnaire after a 7 day interval. Agreement between all 26 items and individual domains of the PCOSQ were examined using the Kappa statistic together with the intra-class correlation coefficient (ICC), for the 26 items, (қ= 0.29–0.69) and for the five domains (қ= 0.30–0.75). The ICC for the domains ranged from 0.78 to 0.96 and significance levels were not reported. It seemed that the time between the first and second administration of the PCOSQ in above articles is unusually short, particularly given that the PCOSQ has a two week recall period.

Bazarganipour *et al.* (14) found the MPCOSQ to have acceptable test–retest reliability when re-administering the questionnaire after a two weeks interval. Agreement between domains of the MPCOSQ was examined using the ICC. The ICC for the domains ranged from 0.71 to 0.92 and these were found to be statistically significant (P<0.05 for all domains).

**3. Content/structural validity**

**a. Content Validity**

Only one study evaluated content validity of the MPCOSQ by expert panels (10 specialists in gynecology and midwifery) (14). The results suggest that the content validity ratio (CVR) for the total scale was 0.92, indicating a satisfactory result. The content validity index (CVI) for the scale was found to be 0.96 suggesting that it had a good content validity.

**b. Factorial validity**

Structural validity of PCOSQ/MPCOSQ has also been evaluated using factor analysis in a small number of studies with inconsistent findings. Some studies have supported the PCOSQ as a five dimensional scale (emotion, hirsutism, infertility, weight, and menstruation domains) (3-4), while others suggest it has six domains (emotion, hirsutism, infertility, weight, and menstruation domains and sixth domain related menstruation) (15). Related with MPCOSQ, Barnard *et al.* (6) reported a seven dimensional scale (emotion, hirsutism, weight, infertility, menstrual symptoms, menstrual predictability and acne), conversely. Bazarganipour *et al.* (14) suggest that it has six domains (emotion, hirsutism, weight concerns, infertility, menstruation and acne).

The discrepancies are: (a) ‘fear of getting cancer’ which loaded on the infertility factor in the Jones *et al.* (4) research and did not load on any factor in Jedel *et al.* (15) study; (b) ‘easily tiring’ which loaded on the weight factor in the Barnard *et al.* (4) research and did not load on any factor in Jedel *et al. *(15) study; (c) ‘Feel sadness because of infertility problems’ which loaded on the emotion factor in the Guyatt *et al*. (3) research; (d) ‘Feel lack of control over PCOS’ which loaded on the menstruation factor in the Guyatt *et al*. (3) research and on emotion factor in Jones *et al.* (4) and Jedel *et al*. (15) studies; (e) ‘late menstrual period’ which loaded on the menstruation factor in the Bazarganipour *et al*. (14) research that this change is approved by confirmatory factor analysis moreover exploratory factor analysis; (f) ‘Irregular menstrual periods’ which not loaded on the menstruation factor in the in Jones *et al*. (4), Barnard *et al*. (6) and Jedel et al. (15) studies and together with ‘late menstrual period’ constructed another factor; (g) ‘headaches’ did not load on any factor in Jedel *et al*. (15) study.

**4. Construct/Criterion validity**

**a. Known-groups validity**

Known-groups validity studies have shown that the PCOSQ/MPCOSQ can differentiate between different populations or symptoms levels. Specifically, the questionnaires are able to discriminate between different populations such as (a) BMI status (3, 4); (b) reproductive history (5, 17); (c) hirsutism status (2, 3); (d) general health group vs. PCOS patients (16); (e) menstrual cyclicity status (3, 17); (f) acne status (17); (g) hyperandrogenemia (3) and (h) taking anti-androgen medication vs. not taking such medication.

**b. Convergent/Divergent validity**

Convergent/Divergent validity has been established between the PCOSQ/MPCOSQ and a variety of other questionnaires that can be used for patients with PCOS. The emotion subscale of the PCOSQ/MPCOSQ has the significant correlation with the “mental health” and “role emotional” subscales (4, 17) and moreover “mental component summary” (16) of the SF-36 which is consistent with the similarity in their purported constructs. Although adequate convergent validity has been demonstrated for a variety of measures with related constructs, weaker correlations were observed with less similar indices. For example, convergent validity detected between the “mental component summary” score of SF-36 and hirsutism (r=0.32), weight (r=0.51), infertility (r=0.49), and menstruation (r=0.25) of PCOSQ indicates that there is a link between the patients’ perceived QOL and psychological status (18). Barnard *et al*. (6) found there to be moderate significant correlations between scores on the PCOSQ and scores on the Zung depression scale.

Regarding divergent validity, Coffey *et al*. found that hirsutism and weight subscales of the PCOSQ did not correlate significantly with the “physical component summary” score of SF-36 and reported this to demonstrate adequate divergent validity (16).

**C. Longitudinal validity**

The longitudinal validity means relationship between changes scores among similar scales/tests measured on different time points where change is expected that was examined only in one article related with PCOSQ. Guyatt *et al*. (3) tested the longitudinal validity of the PCOSQ by determining the correlations between changes in the clinical parameters (i.e. body hair growth, menstrual cyclicity and hyperandrogenemia) and the domains of the PCOSQ. All correlations between changes in the objective measures of hair growth and the changes in the five PCOSQ domains were negative, e.g., less hair growth was associated with an improvement in HRQOL. The change in the F-G score showed a weak but statistically significant (P<0.01) correlation with changes in the four of the five PCOSQ domains, i.e. hirsutism (r=0.22), weight (r=0.17), infertility (r=0.20), and menstruation (r=0.20). Changes in the proportion of normal menstrual cycles demonstrated a weak correlation with change in the infertility domain (r=0.14, P <0.03) and a moderate correlation with the change in the menstruation domain (r=0.31, P<0.001). Correlations between changes in the free T levels and changes in the five PCOSQ domains were negative e.g., possible association between a decrease in free T levels and improvement in HRQOL; however, all correlations were very weak, with only the association between the changes in the weight domain approaching significance (r=0.15, P<0.025).

**5. Responsiveness/Clinical change**

**a. Responsiveness**

Responsiveness to change (i.e. the ability of the questionnaire to detect an important change even if it is small) was examined only in one study. Guyatt *et al*. (3) found that scores of the PCOSQ were responsive to treatment effects (treatment using insulin sensitizing drugs to treat endocrine abnormalities and infertility improvement). In this study with higher dose treatment groups, more improvements were found in the scores on the infertility, emotion, and menstruation subscales of PCOSQ. 

This indicates that the PCOSQ has good ability to detect change over time. However, no difference was found for scores on the hirsutism and weight subscales.

**b. Minimally Clinical Important Difference (MID)**

None of the articles identified in the review established MID for the PCOSQ/MPCOSQ.

**Table I:** Quality of studies on the psychometrics of the PCOSQ/MPCOSQ

**Evaluation criteria**		**Authors**							
		**McCook ** ***et al*** ** (2009)**	**Bazarganipour ** ***et al *** **(2012)**	**Jedel ** ***et al*** ** (2008)**	**Guyatt ** ***et al*** ** (2004)**	**Jones ** ***et al*** ** (2004)**	**Barnard ** ***et al*** ** (2007)**	**Coffey ** ***et al*** ** (2006)**	**Bazarganipour ** ***et al*** ** (2013)**
**Study question**	1. Was the relevant background work cited to define what is currently known about the measurement properties of measures under study, and the potential contributions of the current research question to informing that knowledge base?	1	2	2	1	2	2	2	2
**Study Design**	2. Were appropriate inclusion/exclusion criteria defined?	2	2	2	2	2	2	2	2
	3. Were specific clinical measurement questions/hypotheses identified?	0	2	2	2	2	1	1	2
	4. Was an appropriate scope of measurement properties considered?	0	1	1	1	1	0	0	0
	5. Was an appropriate sample size used?	1	1	0	1	1	1	0	1
	6. Was appropriate retention/follow-up obtained? (for studies involving retesting; otherwise n/a)	n/a	n/a	n/a	1	n/a	n/a	n/a	n/a
**Measurements**	7. Were specific descriptions provided of the measure under study and the method(s) used to administer it?	2	2	2	2	2	2	2	2
	8. Were standardized procedures used to administer all study measures in a manner that minimized potential sources of error/bias (including the study measure and its comparators)?	2	2	2	2	2	2	2	2
**Analyses**	9. Were analyses conducted for each specific hypothesis or purpose?	1	2	2	2	2	2	2	2
	10. Were appropriate statistical tests performed to obtain point estimates of the measurement properties?	2	2	2	2	2	2	2	2
	11. Were appropriate ancillary analyses done to quantify the confidence in the estimates of the clinical measurement property (Precision/Confidence intervals; benchmark comparisons/ROC curves, alternate forms of analysis like SEM/MID,etc.)?	0	0	0	0	0	0	0	0
**Recommendations**	12. Were clear, specific and accurate conclusions made about the clinical measurement properties; that were associated with appropriate clinical measurement recommendations and supported by the study objectives, analysis and results?	0	1	1	2	1	1	2	2
	Subtotals (of columns 1 and 2)	50	77	72	75	77	68	68	77

Total score (sum of subtotals/24*100); if for a specific paper or topic an item is deemed inappropriate then you can sum of items/2*number of items *100 (sum of items/22)×100; ROC: receiver operating characteristic.

SEM: standard error of the mean.

MID: Minimally Clinical Important Difference.

**Table II:** Summary of studies addressing psychometrics properties of the PCOSQ/MPCOSQ

**Author(s)**	**Setting**	**Sample size**	**Reliability**	**Validity**
Jones et al (2004) PCOSQ	UK	82	α: 0.89 to 0.95 ICC: 0.70 to 0.97	Emotion subscale of the PCOSQ moderately correlated with role emotional and mental health SF-36
Guyatt et al (2004) PCOSQ	Canada, UK, USA	393	ICC: 0.54 to 0.93	Hair growth/ diameter: at baseline, related to the hirsutism and menstruation subscales. Menstrual cyclicity: At baseline, related to infertility subscale of PCOSQ. Hyperandrogenemia (free T levels): at baseline related to hirsutism, weight and infertility subscale of PCOSQ. F-G score: related to emotion and hirsutism. Subscales of PCOSQ at baseline.
Coffey et al (2006) PCOSQ	London	118	Not reported	Significant differences were found on all subscales of PCOSQ between women with PCOS and the general population. Emotion subscale of the PCOSQ moderately correlated with MCS on the SF-36. Hirsutism and Weight subscales of the PCOSQ did not correlate significantly with the SF-36 PCS.
Barnard et al (2007) MPCOSQ	UK	1359	ICC: 0.73	Higher BMI negatively correlated with weight concerns, emotion and menstrual predictability subscales of PCOSQ. The scores on the PCOSQ moderately correlated with scores of zung depression scale
Jedel et al (2008) PCOSQ	Sweden	69	α: 0.78 to 0.96	-
Bazarganipour et al (2012) MPCOSQ	Iran	200	α: 0.71 to 0.92 ICC: 0.76 to 0.92	-
Bazarganipour et al (2013) MPCOSQ	Iran	200	Not reported	Emotion subscale of the MPCOSQ moderately correlated with role emotional and mental health of the SF-36. higher BMI associated with negatively correlated with weight subscale of MPCOSQ. Infertility associated with negatively correlated with infertility subscale of MPCOSQ. Higher hirsutism status associated with negatively correlated with hirsutism subscale of MPCOSQ. menstrual irregularities associated with negatively correlated with menstruation subscale of MPCOSQ. higher of acne severity associated with negatively correlated with acne subscale of MPCOSQ.
McCook et al (2009) PCOSQ	USA	158	ICC: 0.76 to 0.96	-

ICC: Interclass correlation.

PCOSQ: PCOS health-related quality of life questionnaire.

MPCOSQ: modified PCOSQ.

## Discussion

This study synthesized current research in 8 studies addressing the psychometric properties of the PCOSQ/MPCOSQ and was able to provide some clinical recommendations regarding its use, within the limits prescribed by the available evidence. Overall, there is moderate evidence for a spectrum of psychometric properties supporting use of the PCOSQ/MPCOSQ in patients with PCOS. The relative importance of different psychometric properties will vary according to purpose. For example, when using the PCOSQ/MPCOSQ to evaluate clinical change in individual patients, the absolute measurement error and responsiveness should be considered most relevant. Conversely, when using the PCOSQ/MPCOSQ to differentiate different levels of HRQOL, known group validity, a form of discriminative validation that tests differences between known subgroups, would be more important. This review considered English/Persian language publications only; however there are a number of published studies which have used translations of the PCOSQ into other languages include Persian, Swedish and Bulgarian widening the applicability of the PCOSQ to non-English speaking settings. In general, published translations used at least some of the recommended procedures for valid translation and demonstrated equivalence.

Overall readability in the English and all translated versions is deemed to be partial acceptable, although some articles finding support to adding the acne domain to PCOSQ which is a significant symptom of PCOS and have the potential to impact negatively on the QOL of women with PCOS (4). It’s seemed the proposed MPCOSQ is more applicable than PCOSQ. While the MPCOSQ may be considered the “gold standard” among outcome measures related to HRQOL in PCOS, this review suggests further investigation is needed.

Within face validity, the selection of items is a crucial stage, since no form of statistical analysis can make up for badly chosen, or worse, missing items (18). Involving patients in this process is essential, because they are the experts on their own QOL. The selection of items was not properly done for the most of the studies in this review, mostly because patients were not involved in the process. However, face and content validity of the MPCOSQ were assessed in one study through consultation with individuals with relevant expertise and the patients with PCOS in order to generate the content of the questionnaire (14). The results suggesting that the questionnaire items match the test objectives and the impact of PCOS on HRQOL of patients. The PCOSQ/MPCOSQ also demonstrates content/structural validity when its internal consistency was examined. A high Cronbach alpha indicates homogeneity of items and supports the validity of the construct being tested (4-5, 16). Structural validity has also been evaluated using factor analysis in a small number of studies with inconsistent findings. Some studies have supported the PCOSQ/MPCOSQ have an additional 1-dimention (related to menstruation) (6, 15) in addition to the main dimensions (emotion, hirsutism, weight, menstruation, infertility and acne) (2-4, 14) of original and modified version of PCOSQ. It is possible the reason of this inconsistency is that factor analysis of the PCOSQ/MPCOSQ yields different results depending on the sample involved and variety of PCOS symptoms in participants. Further research is needed to examine the consistency of factor structure of MPCOSQ, with special attention to the menstruation domain, among specific populations of PCOS.

In all subscales of the PCOSQ/MPCOSQ there were little floor and ceiling effects. Ceiling and floor effects are more likely with nonspecific instruments because some domains measured were unrelated to the disease being considered (19). The lower rate of ceiling and floor effects may be a consequence of an instrument that is more responsive in clinical setting. However, additional studies of patients with repeated measurements following treatment to evaluate the responsiveness of this instrument have been suggested.

The construct validity of the MPCOSQ had been assessed in the majority of the nine studies using known-groups or convergent/divergent validity (3, 6, 16-17). The PCOSQ appears to have reasonable convergent/divergent and known-groups validity. Stronger correlations were observed between the PCOSQ/MPCOSQ and the other disease-specific and generic objective-measures such as the Zung depression scale and SF-36 questionnaire indicating acceptable their validity.

Responsiveness to clinical change is another important feature of an outcome measure. The limited data on effect sizes and standard response demonstrated that the PCOSQ is able to detect clinically meaningful change resulting from the treatment for PCOS and yielded large effect sizes over a 6 month interval (3). Using a responsive outcome measure will facilitate the detection of moderate treatment effects in clinical research. Although rigorous and comprehensive methods were used for this review, there are some study limitations. Firstly, none of the eight studies assessed all the psychometric properties, making comparisons difficult especially regarding face and content validity and reliability. Another limitation of our review stems from the lack of agreed upon quality criteria for synthesis process for psychometric studies. Neither previous systematic review incorporated critical appraisal. There is no clear method to synthesize the extracted psychometric evidence. In some systematic reviews only high-quality studies are synthesized. However, when evaluating an outcome measure, it is important to see how the instrument performs across different contexts and purposes. We summarized the information on psychometrics and usefulness by adapting and expanding a framework used by others. 

Next limitation in our review is that the scope of our search retrieved full-text papers written in only English. We don’t expect this limitation to have a substantial impact on our results, as the majority of translation and validation articles were printed in English, and we were able to extract data from English abstracts in non-English text. Furthermore, there are no levels of evidence that create clear categories for study quality. Therefore, we rank ordered studies by quality to allow the reader and ourselves a mechanism to place greater emphasis on the findings from high-quality studies.

Considering several points in the quality assessment of the research are important. First, the results of some studies for example Guyatt *et al*. were presented in original article and not have some characterized included setting of patients and so. Second, percentage of participation and missing data was not provided in some studies. However, regarding to not present this, we suppose that the prevalence of participation were 100% without missing data. Third, in some study (internet survey), diagnosis of PCOS were based on patient statement and not physician. It is supposed, the patient was informed of the diagnosis of PCOS based on physician diagnosis and this cannot be an important source of bias.

Despite these limitations, reliability coefficients have reached acceptable benchmarks (4, 14-15). A notable gap in current research is the lack of studies defining the MID. Because the PCOSQ/MPCOSQ is often used for research purposes, this information would help to establish clinically important differences for sample size calculations. Moreover, absolute error is a concern, however, and has not been addressed.

Overall, the PCOSQ/MPCOSQ has a number of features that suggest it has good clinical utility. These include its brevity and the fact that it has been translated into a number of languages. It is important to have outcome measures that can be applied across different cultural or language subgroups. Although this cross-validation is well under way, future studies may focus on whether the patient HRQOL in PCOS varies across the subgroups and how these variations are reflected on the PCOSQ/MPCOSQ responses. It is an important consideration that introducing new instruments into clinical practice and research requires tremendous efforts in translation/cultural adaptation and knowledge translation. For these reasons, any suggestions of change to different instruments or in the PCOSQ/MPCOSQ itself should be balanced with this consideration. The most recent suggestion is that the modify version of PCOSQ (MPCOSQ) is more applicable than PCOSQ and can be considered for assess of HRQOL in PCOS patients in future studies. Perhaps, most importantly, there are gaps in defining clinically useful comparative data and benchmarks. For example, the work on MID is not done, yet.

An extensive search strategy led to the identification of eight studies, considerably more than earlier reviews. These questionnaires were described and rated on their psychometric quality on the basis of clearly defined criteria. Despite identifying nine studies that address at least one psychometric property of the MPCOSQ, a number of issues remain unresolved. These knowledge gaps should be the focus of future research. Our review also raises concerns about the quality of existing studies, because only half of these reached a quality level of more than 75%.

Instrument development is a continuing procedure. The properties such as validity, reliability and responsiveness studied to date are not unchanging properties but specific to the instrument used in specific situation and population. More studies are needed to evaluate the MPCOSQ’ psychometric properties, with special attentions to the factorial validity, test-retest reliability using appropriate statistical measures, and defining the MID.

Clinicians looking for a disease-specific measure for assessing pre- and post-treatment symptom severity can be confident that the MPCOSQ is responsive to change, repeatable over time and that the scales measure what they purport to measure. The recommendation for future development of MPCOSQ presents at below:

1. Future studies on the psychometric properties in different clinical groups are warranted and should specifically state hypotheses that are to be evaluated, including the specific psychometric property being analyzed, as well as the expected outcome.

2. Studies that determine SEM and MID in different subgroups are needed to provide more accurate outcome evaluation.

3. Qualitative studies and cognitive interviewing that evaluate how patients respond to items of the MPCOSQ would inform our understanding of self-reported disability as reflected on the MPCOSQ.

## Conclusion

In summary, the MPCOSQ offers an acceptable patient based outcome measure of HRQOL status for which there is widespread acceptance on content and construct validity, reliability and internal consistency and it should be recommended for inclusion in future trials on PCOS interventions. However, future study related to factor and longitudinal validity, absolute error of measurement, minimal clinically important difference and responsiveness are needed.
